# An Interprofessional Approach to Teaching About Postpartum Depression

**DOI:** 10.1111/jmwh.70056

**Published:** 2025-11-17

**Authors:** Abigail Howe‐Heyman, Joseph Schatz, Marissa DeCesaris Siegel

**Affiliations:** ^1^ Department of Family and Community Health University of Pennsylvania School of Nursing Philadelphia Pennsylvania

**Keywords:** depression, interprofessional education, mental health, midwifery, nurse practitioners, postpartum, psychiatric, women's health

## Abstract

**Introduction:**

Mental health conditions are the leading cause of pregnancy‐related death in the United States. The purpose of this study was to explore the use of interprofessional simulation for postpartum depression management.

**Methods:**

Midwifery, women's health gender‐related nurse practitioner, and psychiatric mental health nurse practitioner students participated in an interprofessional postpartum depression simulation. Full‐time students enrolled at the university in 2024 and 2025 were eligible to participate in a study using a pre‐posttest design. Attitudes toward and confidence in management of postpartum depression were assessed before and after the simulation using a modified version of the Revised Depression Attitude Questionnaire (R‐DAQ).

**Results:**

There was a significant increase in modified R‐DAQ scores following the intervention. Students’ comments showed that they found the experience positive and that it helped improve their interprofessional communication and confidence in the management of postpartum depression.

**Discussion:**

This is the first known study assessing the efficacy of an interprofessional postpartum depression simulation. Limitations include a lack of longitudinal data, a single site, and the use of a modified rating scale. Interprofessional simulation can be an effective educational intervention to prepare advanced practice nursing students to manage postpartum depression.

## INTRODUCTION

Perinatal depression is a mood disorder marked by the onset of depressive symptoms in pregnancy or up to one year after birth.^1^ Major depressive disorder with peripartum onset, as defined by the *Diagnostic and Statistical Manual of Mental Disorders, Fifth Edition*, may be diagnosed during pregnancy or up to 12 months after giving birth.^2^ Approximately 14% of postpartum individuals in the United States are diagnosed with postpartum depression (PPD), and it is likely that significantly more postpartum people are affected by the condition, as PPD is frequently unreported, underdiagnosed, or untreated.^3^ Mental health conditions, including perinatal depression, are the most common underlying cause of pregnancy‐related deaths in the United States, accounting for 22.5% of pregnancy‐related deaths.^4^ Perinatal depression is characterized by persistent feelings of sadness, anxiety, and loss of interest in activities.^1^ People experiencing PPD often have highly negative and persistent ruminations about their capacity to care for their child and guilt that their actions may have a negative impact on the infant.^5^

  

Continuing education (CE) is available for this article. To obtain CE online, please visit http://www.jmwhce.org. A CE form that includes the test questions is available in the print edition of this issue.

For the birthing person, untreated PPD is associated with a higher risk of substance use and suicide.^6^, ^7^, ^8^ For the family, untreated PPD can lead to infant maltreatment,^9^ relationship challenges,^10^ and less optimal parent‐infant interactions.^11^ And for the infant, untreated PPD in a birthing parent is associated with lower rates of breastfeeding,^12^ higher rates of infant and childhood illness,^13^ hospitalizations,^14^ and cognitive, emotional, and behavioral problems.^15^, ^16^ Universal screening of postpartum people for perinatal depression and anxiety disorders using a standardized, validated instrument is recommended by American College of Obstetricians and Gynecologists, American College of Nurse‐Midwives, American Psychological Association, and US Preventive Services Task Force.^17^, ^18^, ^19^, ^20^ A commonly used validated tool is the Edinburgh Postnatal Depression Scale (EPDS). The EPDS is a self‐administered 10‐item questionnaire with a sensitivity of 55% to 98% and specificity of 68% to 97%. Scores on the EPDS range from 0 to 30.^21^ A positive screen (score ≥10) or a positive result for the suicidality item should be followed up with immediate further mental health assessment.^22^ The recommended first‐line treatment for mild PPD is psychotherapy. Pharmacotherapy is recommended for patients experiencing moderate to severe PPD, or for patients who cannot access or are not willing to participate in psychotherapy.^2^, ^23^ Importantly, prior to initiating pharmacotherapy, clinicians should screen patients for a prior history of mania, as treating bipolar disorder with antidepressant monotherapy can precipitate mania or psychosis and increase the risk of suicide and infanticide.^2^

**QUICK POINTS**

✦ Peripartum mental health issues are common and can result in pregnancy‐related death.
✦ Postpartum depression may be underrecognized and undertreated, in part due to provider discomfort managing this condition.
✦ Advanced practice nursing students require training in interprofessional communication and collaboration.
✦ Interprofessional postpartum depression simulation is a novel method for improving student attitudes toward and confidence in managing postpartum depression.

As clinicians who provide care to postpartum patients, midwives and women's health gender‐related nurse practitioners (WHGRNPs) are well‐positioned to diagnose and treat PPD. However, a significant barrier for many midwives and WHGRNPs in the management of PPD is a lack of training in the diagnosis and management, leading clinicians to feel unprepared to care for these patients.^24^, ^25^, ^26^, ^27^ As providers of mental health care in the primary care setting, psychiatric mental health nurse practitioners (PMHNPs) are also skilled in the care of patients with PPD, although challenges with timely access to appointments, provider shortages, and stigma are often significant barriers to accessing care.^28^, ^29^ When nurses and midwives have access to collaborative practice with other health care providers, they are more likely to feel comfortable caring for patients with PPD.^24^, ^26^, ^30^ The primary care behavioral health model of care, in which perinatal mental health and clinical care are provided side‐by‐side in an interprofessional setting, is an evidence‐based approach to providing wraparound care to patients.^21^, ^31^, ^32^, ^33^, ^34^

To address clinician comfort with the diagnosis and management of PPD, we developed a simulation learning experience to teach PMHNP, WHGRNP, and midwifery students to collaboratively diagnose and manage PPD. Our main learning objective of this simulation experience was to have students collaborate across professional identities to develop a safe plan of care for a patient with PPD, with a goal of increasing comfort with the diagnosis and management of this condition. We also sought to expose students to interprofessional communication and management through exposure to the primary care behavioral health model of care. Interprofessional communication has been identified as a key component of high quality, safe clinical care.^34^, ^35^, ^36^, ^37^ Because of the importance of interprofessional care, interprofessional partnerships are a required domain in the American Association of Colleges of Nursing (AACN) *The Essentials: Core Competencies for Professional Nursing Education* ^38^ and a required criterion for accreditation of midwifery education programs by the Accreditation Commission for Midwifery Education (ACME).^39^ Therefore, our secondary learning objectives were that students would practice communicating across disciplines and with their patients about the diagnosis and management of PPD, and students would describe the benefits of the primary care behavioral health model of care. We also had specific learning objectives for the WHGRNP and midwifery students and the PMHNP students (Table 1). The purpose of this study was to identify whether this simulation learning experience increased PMHNP, WHGRNP, and midwifery students’ comfort with the diagnosis and management of PPD.

**Table 1:** Interprofessional Postpartum Depression Simulation Learning Objectives

**Primary learning objectives for all learners**
Collaborate in developing a safe plan of care for a patient with postpartum depression
Practice communicating across disciplines and with their patient about the diagnosis and management of PPD
Describe the benefits of the primary care behavioral health model of care
**Learning objectives for PMHNP students**
Apply behavioral activation using a person‐centered approach with a patient experiencing postpartum depression
Demonstrate an effective screening for the current history of mania and postpartum psychosis
Discuss risks vs benefits with a colleague regarding initiating an antidepressant when the infant is breast or chestfeeding
**Learning objectives for WHGRNP and midwifery students**
Consider the value of nonpharmacologic interventions when supporting patients with postpartum depression
Discuss the importance of screening for a history of mania prior to starting a patient on an antidepressant
Practice initiating an antidepressant for a patient with postpartum depression

Abbreviations: PMHNP, psychiatric mental health nurse practitioner; PPD, postpartum depression; WHGRNP, women's health gender‐related nurse practitioner.

## METHODS

### Participants

Participants were midwifery, WHGRNP, and PMHNP students at a private university in the Northeast. All 99 students enrolled in clinical coursework in 2024 and 2025 were eligible to participate (student nurse‐midwife and WHGRNP, 36; PMHNP, 63). Participation was voluntary and anonymous—informed consent was provided through a written statement, and participants were given the option to opt out. This study was deemed exempt from review by the University of Pennsylvania Institutional Review Board. The study team consisted of 3 doctorally prepared faculty members, one of whom is a nurse‐midwife and 2 of whom are PMHNPs.

### Intervention

Midwifery and WHGRNP students are co‐enrolled in the postpartum care course and so are considered as one group for the purposes of this study. All PMHNP, WHGRNP, and midwifery students received a synchronous lecture on peripartum mental health and watched an asynchronous lecture socializing them to the primary care behavioral health model of integrated care. The synchronous lecture focused on assessment and management of peripartum mental health disorders including both pharmacologic and nonpharmacologic treatment. Nonpharmacologic treatment focused on recommendations that can be made by midwives and nurse practitioners such as behavioral activation, a common technique for managing depression that involves encouraging patients to engage in meaningful or pleasurable activities.

Prior to the start of the simulation, students were given a patient chart to review (Supporting Information: Appendix S1). The patient scenario described a 6‐week postpartum patient who had a cesarean birth under general anesthesia, limited social support, and an EPDS score of 13 of 30 on postpartum day 2, all factors that increase the patient's likelihood of a postpartum mood disorder. The chart also included a current EPDS with a score of 22 of 30 and an Abuse Assessment Screen^40^ with a score of 0 of 5. The patient documents did not endorse feelings of postpartum anxiety, suicidality, or intimate partner violence, and this was not the focus of the simulation.

**Figure 1:**
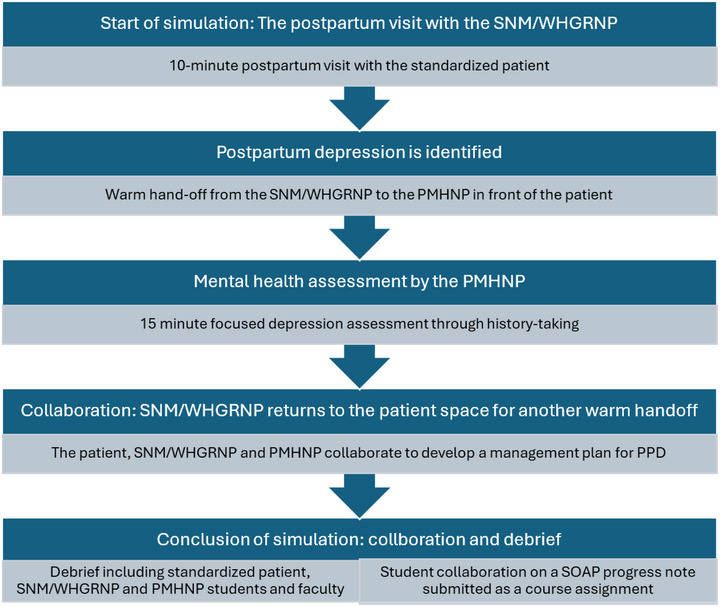
Flow of Simulation Abbreviations: PMHNP, psychiatric mental health nurse practitioner; PPD, postpartum depression; SNM, student nurse‐midwife; SOAP, subjective, objective, assessment, plan; WHGRNP, women's health gender‐related nurse practitioner.

The standardized patient was played by a trained patient communicator from a locally recognized standardized patient agency. On the day of the simulation, there was a prebrief where the students introduced themselves to their interprofessional partner and the faculty set expectations. The flow of the simulation (Figure 1) was as follows: (1) the midwifery or WHGRNP student spent 10 minutes with the standardized patient for a postpartum follow‐up visit and identified PPD; (2) the midwifery or WHGRNP student provided a verbal warm handoff to the PMHNP student in front of the patient; (3) the PMHNP student completed a focused depression assessment and encouraged behavioral activation; (4) the PMHNP returned the warm handoff to the midwifery student in front of the patient; (5) the midwifery or WHGRNP and PMHNP student articulated a plan to the patient including a prescription medication; (6) a debrief occurred with the students, standardized patient, and interprofessional faculty; and finally (7) the students collaborated in writing a Subjective, Objective, Assessment and Plan progress note that focused only on the assessment and plan sections and which was submitted to faculty for feedback. These steps were based on the International Nursing Association for Clinical Simulation and Learning's *Healthcare Simulation Standards of Best Practice: Simulation‐Enhanced Interprofessional Education*.^41^

### Design

This study used a pretest‐posttest design to measure changes in attitudes toward PPD following an interprofessional simulation experience. Participants created a participant ID number and were able to complete the pretest within the week prior to the simulation. The pretest consisted of a modified version of the Revised Depression Attitude Questionnaire (R‐DAQ).^42^ Following the simulation experience, study participants had one week to complete the posttest, which consisted of the modified R‐DAQ and 2 open‐ended questions related to interprofessional communication and self‐reflection on attitudes toward mental health. Pretest and posttest data were matched by participant ID number, which participants needed to remember in the time between the pretest and posttest.

### Outcome Measurement

The R‐DAQ is a 22‐item instrument designed to assess health care professionals’ attitudes toward depression.^42^ Developed to address limitations in the original Depression Attitude Questionnaire (DAQ), the R‐DAQ evaluates 3 key dimensions: professional confidence in managing depression, therapeutic optimism or pessimism regarding treatment outcomes, and a generalist perspective on the integration of depression care into general health care practice. Each item is rated on a 5‐point Likert‐type scale, with higher scores indicating more positive attitudes toward depression care. The R‐DAQ has demonstrated good internal consistency, with a Cronbach's α coefficient of .84, and satisfactory test‐retest reliability, with an intraclass correlation coefficient of 0.62.^42^ The tool's factor structure has been validated in various health care settings.^43^, ^44^ The R‐DAQ was adjusted, with the author's permission, to meet the needs of this simulation, with “depression” shifted to “postpartum depression.” Two items from the R‐DAQ, “becoming depressed is a natural part of adolescence” and “becoming depressed is a natural part of being old,” were replaced with the single item “becoming depressed is a natural part of having a baby.” With a total of 21 items, possible total scores ranged from 21 to 105.

### Data Collection and Analysis

All data were collected anonymously through Qualtrics software, with no identifying data included. Data were analyzed using IBM SPSS Statistics (v.28.0.0.0). Descriptive statistics were used to identify mean scores and SDs. Paired *t* tests were used to analyze changes in R‐DAQ score from pre‐ to posttest using the participant ID number. Independent *t* tests were used to assess differences between midwifery and WHGRNP versus PMHNP specialties. The Transparent Reporting of Evaluations with Nonrandomized Designs reporting guidelines were used to guide study design and manuscript preparation (Supporting Information: Appendix S2).^45^ Participants also answered open‐ended questions regarding their experience. These comments were reviewed for overall trends and key ideas, but a formal qualitative analytic method was not used to analyze these comments.

All team members identify as White and primarily work with underserved communities. One member has experience in integrated care through work as a PMHNP in a federally qualified health center, and another has completed specialized training in perinatal mental health. All team members have been involved in efforts to improve diversity and inclusivity training for students in their respective programs, particularly with regard to race, gender identity, and sexual orientation. Throughout the study, we reflected on the power dynamics between faculty and students and made efforts to emphasize the optional nature of participation in the research of this classroom activity.

## RESULTS

Out of 99 eligible students in the 2024 and 2025 cohorts, 94 (94.9%) consented to participate in the study and completed the pretest survey, and 90 (90.9%) students completed the posttest survey. Cronbach's α for the modified R‐DAQ was 70. For unpaired data (Table 2), pretest R‐DAQ scores ranged from 70 to 102, with a mean (SD) score of 90.52 (6.66). Posttest scores ranged from 67 to 105, with a mean (SD) score of 96.09 (7.18). There was a significant increase in posttest scores compared with pretest scores (*P* <.001). There were no significant differences between specialties on pretest or posttest scores and no significant difference in the percentage of eligible students who elected to participate between specialties. There were 33 participants who did not enter a matching participant ID for the posttest, leaving a total of 61 responses that were eligible for comparison of individual score changes between pretest and posttest. Of these 61 participants, 35 (57.4%) were in the PMHNP program. There was no significant difference in the modified R‐DAQ mean score between paired and unpaired data sets (*P* = .663).

**Table 2:** Unpaired Scores of the Modified Revised Depression Attitude Questionnaire

	Pretest		Posttest	
Program	n	Mean (SD)	n	Mean (SD)
SNM and WHGRNP	37	89.38 (6.34)	31	91.26 (6.83)
PMHNP	57	94.16 (7.40)	57	97.10 (6.91)
Total	94	90.52 (6.67)	90	96.09 (7.17)

Abbreviations: PMHNP, psychiatric mental health nurse practitioner; SNM, student nurse‐midwife; WHGRNP, women's health gender‐related nurse practitioner.

For paired data (Table 3), pretest scores ranged from 71 to 102 with a mean (SD) score of 91.00 (6.66). Posttest scores ranged from 67 to 105 with a mean (SD) score of 96.39 (7.04). The mean (SD) change from pretest to posttest was 5.39 (6.96), which was statistically significant (*P* <.001; 95% CI, 3.61‐7.18) with a moderate effect size (Cohen's *d* = 0.775). There were no significant differences between specialties on pretest, posttest, or change scores. Regarding subscales, all showed an increase from pretest to posttest, but only the professional confidence in depression care subscale reached statistical significance (*P* <.001; 95% CI, 3.12‐4.85). Looking at individual items, the 3 items with the highest change between pretest and posttest were from the professional confidence subscale: “I feel comfortable in dealing with the needs of patients with postpartum depression,” “I feel confident in assessing depression in postpartum patients,” and “I feel confident in assessing suicide risk in patients presenting with postpartum depression.”

**Table 3:** Paired Modified Revised Depression Attitude Questionnaire Change Scores (n = 61)

	Change Score	
Measure	Mean (SD)	*P* Value
Modified R‐DAQ total	5.39 (6.96)	<.001
Professional confidence subscale	3.98 (3.37)	<.001
Therapeutic optimism subscale	0.49 (3.90)	.328
Generalist perspective subscale	0.72 (1.86)	.004

Abbreviation: R‐DAQ, Revised Depression Attitude Questionnaire

Review of open‐ended responses on the posttest survey showed students enjoyed the simulation and felt overall that their interprofessional communication was positive. Areas of self‐improvement noted by students included more consistent use of pronouns and improved flow between providers and better role delineation, particularly regarding treatment plan and follow‐up. Students reported feeling more confident working with patients outside of their specialty, with several noting this was their first exposure to integrated care. Students also identified barriers to implementing regular integrated care for PPD in clinical practice, such as short visit duration and limited mental health provider availability. Sample quotes provided in Table 4 demonstrate the strengths and limitations of this simulation.

**Table 4:** Free‐Text Statements From Students Regarding the Simulation Experience

Sample Quotes
“I think I was initially scared of postpartum patients; this sim helped me build confidence in my ability to care for this population.” Participant ID: 641 (PMHNP)
“I previously only thought of integrated care within the context of primary care, but after the sim, I think this model should also be applied to WH and OBGYN clinics.” Participant ID:160 (PMHNP)
“I didn't have much experience with PPD during clinical so far, so being able to practice having hard conversations was a great experience.” Participant ID: 500 (SNM/WHGRNP)
“After participating in this simulation, I have noticed that patients with postpartum‐related mental health issues may not fully acknowledge their struggles with mental health challenges. They may view their symptoms as normal. Therefore, I will ensure that I educate my future postpartum patients about postpartum mental health problems, making them aware that experiencing mental health symptoms as a new mother is not typical.” Participant ID: 671 (SNM/WHGRNP)
“I think my main takeaway from this simulation is that individuals who are in the postpartum period may not have the energy or time to be able to engage in nonpharmacological therapeutic interventions, even if they know it could help them.” Participant ID: 178 (PMHNP)
“I felt empowered as a burgeoning provider to have a care partner there, and that together we made for a stronger team that ultimately benefited the patient all the more.” Participant ID: 765 (PMHNP)

Abbreviations: OBGYN, obstetrics‐gynecology; PMHNP, psychiatric mental health nurse practitioner; PPD, postpartum depression; SNM, student nurse‐midwife; WH, women's health; WHGRNP, women's health gender‐related nurse practitioner

## DISCUSSION

Participation in an interprofessional simulation improved midwifery, WHGRNP, and PMHNP students’ attitudes toward PPD and its treatment. The simulation experience also improved students’ confidence in the assessment and management of PPD. Literature has demonstrated that participation in scenario‐based simulation experiences provides students with practical learning in a safe environment that translates into sustained changes in clinical practice.^46^ Although we were not able to demonstrate sustained changes in attitudes and practice based on this intervention, we anticipate that participants will feel more confident managing PPD in the clinical setting upon graduation than they would have without the experience. Given that one of the greatest barriers to care for patients with PPD is access to providers who are supportive and comfortable with the management of the condition, training that improves provider confidence has the potential to improve care for patients with PPD.^47^, ^48^, ^49^

In their free‐text statements, students reported improvement in interprofessional communication skills with a desire for more practice with similar scenarios. The benefits of interprofessional education translate to future clinical practice and include reduced frequency of communication breakdowns, improved morale, enhanced professional confidence, promotion of mutual understanding between professions, improved interprofessional communication, and reflective practice.^50^ Considering the impact on patient safety^36^, ^37^ and provider satisfaction^50^ that is derived from interprofessional and interprofessional collaboration, this simulation has the potential to influence patient care and safety across settings in the future.

Competency‐based education (CBE) is a form of curriculum that emphasizes students’ demonstration of measurable skills and practical experience.^51^, ^52^, ^53^ This approach to learning is supported by ACME and AACN.^38^, ^39^ Educational methods are student‐centered and involve active learning strategies such as simulation. Although much of CBE focuses on individually measured skills, effective teamwork is necessary for nurse competency. This allowed students to focus on cognitive and affective domains of learning. The use of standardized patients supported the development of communication skills and made the scenario more realistic to clinical practice. This simulation also aligns with AACN *Essentials* Domain 10, Personal, Professional, and Leadership Development, by debriefing immediately after scenario completion and the flexibility required to interact with patients with complex needs outside of one's own specialty.^54^

### Strengths

This is the first study that we are aware of exploring the use of an interprofessional PPD simulation with midwifery, WHGRNP, and PMHNP students. This is also the first study examining changes in modified R‐DAQ scores following a targeted intervention. More than 90% of eligible students participated in the study in both cohorts, and the study team had department support for interprofessional education simulation.

### Limitations

This study had several limitations, including lack of control group and small sample size for paired analysis. Both cohorts had higher numbers of PMHNP students than midwifery and WHGRNP students. Follow‐up was limited to a single time point shortly after intervention delivery; therefore, it is unknown if positive changes persist over time. It is also difficult to determine the correlation between the modified R‐DAQ score and competency in clinical practice. With baseline modified R‐DAQ scores being high, there is a possible ceiling effect, making it difficult to see significant score changes at posttest. Participants were limited to students at a private university in a large urban area. The pretest modified R‐DAQ was administered after the didactic component of the intervention, which could partially explain the high baseline scores. An untested modified version of the R‐DAQ was used in this study; however, the calculated Cronbach's α of.70 shows good reliability. Additionally, most studies using the original Depression Attitude Questionnaire and R‐DAQ were conducted outside of the United States and with practicing clinicians rather than health care students. Due to the dearth of existing research using the modified R‐DAQ, scores from this study cannot be compared to previous work. A large number of students did not enter a matching participant ID for the posttest, reducing the sample size for paired *t* tests. A formal qualitative analytic method was not used to review students’ open‐ended feedback, and this may have led to an incomplete or inaccurate interpretation of their opinions. In future research, students will be encouraged to use the last 3 digits of their phone number as their participant ID to increase matching pairs without compromising participant anonymity.

### Implications for Education

This is a teaching modality that could be used by others to help advanced practice nursing students meet level 2 competencies and subcompetencies for AACN's interprofessional partnership domain and ACME's criteria IV related to curriculum. Simulation has been shown to be an effective format for interprofessional education.^41^ Interprofessional simulation can be a relatively cost‐effective education tool as individual program costs are reduced by sharing standardized patient costs among students from multiple specialties; this simulation cost $61 per student. Costs included the use of the simulation laboratory and the hiring of standardized patients, who were employed by an outside company specializing in educating clinicians about communication. Because the simulation was integrated into clinical coursework for midwifery, WHGRNP, and PMHNP students, there was no additional cost for faculty staffing. We have also considered ways to reduce costs by using alumni and community members as our standardized patients in the future. Interprofessional communication and collaboration can improve quality and safety of care for patients.^55^, ^56^ Midwifery, WHGRNP, and PMHNP faculty involved in the facilitation of the simulation also reported high levels of support for interprofessional experiences.

### Implications for Clinical Practice

Although this study did not measure the prolonged impact of the simulation on clinical practice, other researchers have found that participating in simulation education does have a lasting influence on clinical practice, especially around clinician self‐confidence in recognizing and managing conditions that they have worked with in the simulation environment.^57^, ^58^ Increased use of this teaching modality may help to expand the number of clinicians who are comfortable diagnosing and managing PPD, addressing one of the leading barriers for midwives, WHGRNPs, and PMHNPs in the management of PPD.^24^, ^25^, ^26^, ^27^, ^28^, ^29^ Expanding the workforce that is trained and confident in the management of PPD has the potential to improve access to care for patients with PPD.

### Suggestions for Future Research

Further evaluation of the psychometric properties of the modified R‐DAQ used in this study could validate its use in assessing attitudes toward PPD. Longitudinal follow‐up could also help determine the prolonged impact of this intervention on attitudes toward PPD by using the modified R‐DAQ with clinicians who did and did not participate in the simulation. Future iterations of this simulation may include gathering formal faculty feedback or adapting the scenario to additional perinatal mental health concerns, such as postpartum anxiety or psychosis. Students also expressed an interest in learning more about birth trauma and its influence on the development of PPD.

## CONCLUSION

Scenario‐based simulation in an interprofessional setting improved students’ confidence in the management of PPD. Students across disciplines also reported that they enjoyed collaborating with students outside of their specialty. Use of this educational activity has the potential to positively influence future clinicians’ practice and the care that they provide to patients in the important and often underserved postpartum period.

## CONFLICT OF INTEREST

The authors have no conflicts of interest to disclose.

## Supporting information




**Appendix S1**. Simulation Teaching Materials

**Appendix S2**. Transparent Reporting of Evaluations With Nonrandomized Designs (TREND) Checklist
