# Supplementary material for: An Interprofessional Approach to Teaching About Postpartum Depression
Source: J Midwifery Womens Health. 2025 Nov 17;71(2):204–11. doi: 10.1111/jmwh.70056 (PMC13067922; doi:10.1111/jmwh.70056)
Supplement: Supplementary file 1 — Appendix S1. Simulation Teaching Materials [file JMWH-71-204-s001.pdf]

Scenario

Kendall Smith is a 32yo single parent presenting for 3-week postpartum follow up visit. Kendall had a difficult birth experience- they underwent a multi-day induction, got to fully dilated, and pushed for two hours After two hours, the fetus had a prolonged fetal heart deceleration that led to an emergency cesarean birth under general anesthesia. The infant, Frankie, was admitted to the NICU for three days with respiratory distress. Kendall and Frankie had a difficult time initiating lactation, as they were separated for 24 hours and then had limited time together during the first few days.

Today, Kendall reports that they have been having a difficult time since the birth. After working with a lactation consultant, they are successfully breast/chestfeeding. Frankie is on a normal growth curve. However, Frankie cries a lot and the pediatrician has described him as "colicky." On observation, the infant is hard to settle/crying throughout exam.

Kendall reports that they are getting a cumulative six hours of sleep per day. They report feeling fatigue, frustration, low appetite, and a hard time engaging in usual self-care. They are estranged from their family and have been relying on friends to bring in groceries and help with household chores. They are not enjoying things that they normally would. They are self-critical and feeling helpless/hopeless

Prior to meeting with the patient, Edinburgh is scored by the medical assistant at 22. Chart review shows appropriate growth/on curve. IPV screening negative.

Flow of Simulation

Chart review: Pre-sim

- Edinburgh
- IPV screening

Part 1: WH/MW Intervention (10 min)

- Assessment of baby (growth, sleep, feeding, output, behavior)
- Assessment of parent (adjustment, coping, social support, family adjustment to newborn, confidence in newborn care)

Part 2: Warm-handoff (5 min)

- Reason for referral (in front of patient)

Part 3: PMHNP Intervention (15 min)

- History
- Safety evaluation (SI, HI, s/sx psychosis and mania)
- Non-pharm intervention

Part 4: Warm-handoff (5 min)

- Plan (pharm intervention)

Part 5: Debrief with SP (5 min)

Part 6: Debrief with IPE small group

Supplies:

- Baby
- Crying noises
- Completed Edinburgh

Interdisciplinary Postpartum Depression Simulation

Learning Objectives:

WH/CNM:

- Consider the value of non-pharmacologic interventions when supporting patients with postpartum depression
- Discuss the importance of screening for a history of mania prior to starting a patient on an antidepressant
- Practice initiating an antidepressant for a patient with postpartum depression

PMHNP:

- Apply behavioral activation using a person-centered approach with a patient experiencing postpartum depression
- Demonstrate an effective screening for current/history of mania and postpartum psychosis
- Discuss risks vs benefits with a colleague regarding initiating an antidepressant when the infant is breast/chestfeeding

Both:

- Describe the benefits of the primary care behavioral health model of care
- Practice a warm-handoff
- Collaborate in developing a safe plan of care for a patient with a postpartum depression

Simulation Assignment:

Prework:

- Watch the "Postpartum Depression/Integrated Care" recorded lecture
- Review at least one clinical practice guideline regarding perinatal depression, postpartum depression, or general management of depression
- Review the "Intake Sheet," depression screener, and IPV screener
- Complete pretest

Simulation:

- Complete the simulation keeping to the "Flow of simulation" (next page)

Post-simulation:

- Collaborate with interdisciplinary partner to finalize your plan. Your plan can be different from the plan you verbalized during the simulation.
- Write up the Plan and submit to your course faculty
- Complete posttest

Flow of Simulation

Part 1: Introductions/ ice breaker (5 min)

- Your name, your specialty
- For PMHNP students: what- if any- is your personal or professional experience with perinatal care?
- For MID/ WHGRNP: what- if any is your personal or professional experience with mental health care/

Part 2: WH/MW Intervention (10 min)

- Assessment of baby (growth, sleep, feeding, output, behavior)
- Assessment of parent (adjustment, coping, social support, family adjustment to newborn, confidence in newborn care)

Part 3: Warm-handoff (5 min)

- Reason for referral (in front of patient)

Part 4: PMHNP Intervention (15 min)

- History
- Safety evaluation (SI, HI, s/sx psychosis and mania)
- Non-pharm intervention

Part 5: Warm-handoff (5 min)

- Plan (pharm intervention)

Part 6: Debrief (10 min)

- PMHNP student- how did it feel? What went well, what would you do differently?
- MID/WHGRNP student- how did it feel? What went well, what would you do differently?
- SP: communication feedback
- PMHNP faculty: clinical feedback
- MID/WHGRNP faculty: clinical feedback

INTAKE SHEET

Kendall Smith (they/them) is a 32 yo G2 P1011 3 weeks postpartum being seen at 3 weeks for a mid-postpartum visit. It is standard practice at your office that all postpartum patients have a mid-postpartum visit.

OB history:

- C/S three weeks ago after 3-day induction. Got to FD, pushed for two hours, had STAT C/S under general anesthesia for prolonged FHR decel.
    - Uncomplicated pregnancy- iron-deficiency anemia managed with diet
    - Baby boy, Frankie, spent 3 days in the NICU for RDS. +breast/chestfeeding after initial difficulty
    - Had an Edinburgh score of 13 on PP day 2
- Hx. Of SAB at 6 weeks at age 22.

Prior hx:

- PMHx: exercise-induced asthma- uses albuterol pump prn
- PSHx: C/S three weeks ago, no other surgeries
- Fam Hx: pt is adopted and unaware of biological Fam Hx
- Psych/MH Hx
    - History of depression as a teen.
    - Was in therapy for ~ 6 mos- didn't like therapist so stopped going
    - Took meds (unsure which?) for 3 years, then self-d/c'd. Helped.
    - No hospitalizations, no SI/SA, no HI/HA
- Social Hx
    - Lives alone with infant, Frankie
    - Conceived by anonymous sperm donor- intended single parent
    - Estranged from family. Support network is friends in the area
    - No hx of IPV or sexual abuse
    - Substance use
        - Vaped 1 x/ day prior to pregnancy
        - Alcohol: 1-2 beer/wine nightly prior to pregnancy
        - Drugs: used marijuana 1 x/ week prior to pregnancy

VS by MA today

- BP: 113/72
- P: 68
- T: 98.2
- R: 16

Physical exam deferred

SOA(P) Note Instructions

The (S) and (O) sections have been completed for you below. Please write the (A) and (P) sections. Do not exceed one page.

This assignment is worth three points and should be submitted via Canvas.

Rubric:

	Missing or Unsafe (0)	Present/Impractical (50%)	Present/Practical (100%)
Non-pharm Recs (1 pt)			
Pharm Recs (1 pt)			
Follow-up Plan (1 pt)			

Claire Fagin Health Center

Patient: Kendall Smith

DOS: 04/18/2023

Provider:

C/C: depression

Subjective: Consulted by WHNP for positive depression screen. Patient is a 32yo single parent, 3 weeks s/p emergent c-section. Difficult delivery. Baby Frankie is colicky and sleeping poorly. Kendall is presenting with depressed mood, low motivation, low energy, tendency towards isolation, self-critical rumination, anhedonia, and lower frustration tolerance. Symptoms present for past 10+ days. Is allowing family to provide help, but not interested in engaging with them otherwise. Feeling overwhelmed in transition to parent of newborn. No current/history of s/sx SI/HI/psychosis/mania. History of moderate depression in high school and took unknown antidepressant for 3+ years with good response. IPV screen negative.

Objective: Edinburgh = 22. Presents as tired, poor eye-contact, low volume, often 1-2 word responses but eventually longer responses. Mood depressed with constricted affect. Thinking is linear and goal-directed. No SI. No AVH. Fair insight. Fair judgment. Good impulse control.

Assessment:

Plan:

Abuse Assessment Screen (AAS)

1. Have you ever been emotionally or physically abused by your partner or someone important to you?

   a. Yes

   ✓ b. No

2. Within the last year, have you been hit, slapped, kicked or otherwise physically hurt by someone?

   a. Yes

   ✓ b. No

   If yes, by whom? (Circle all that apply)

   1. Husband
   2. Ex-husband
   3. Boyfriend
   4. Stranger
   5. Others (specify)___________

   Number of times _______

3. Since you have been pregnant, have you been hit, slapped, kicked or otherwise physically hurt by someone?

   a. Yes

   ✓ b. No

   If yes, by whom? (Circle all that apply)

   1. Husband

2. Ex-husband

3. Boyfriend

4. Stranger

5. Others (specify)

Number of times _______

Indicate the area of injury:____________________________

Score the most severe incident to the following scale:

1. Threats of abuse, including use of a weapon

2. Slapping, pushing; no injuries and/or lasting pain

3. Punching, kicking, bruises, cuts and/or continuing pain

4. Beaten up, severe contusions, burns, broken bones

5. Head, internal, and/or permanent injury

6. Use of weapon, wound from weapon

4. Within the past year, has anyone forced you to have sexual activities?

a. Yes

✓ b. No

If yes, by whom?

1. Husband

2. Ex-husband

3. Boyfriend

4. Stranger

5. Others (specify)

Number of times _______

5. Are you afraid of your partner or anyone you listed above?

   a. Yes

   ✓ b. No

6. Do you want us to reveal this information to: (for those who answered yes to questions 2,3, or 4) N/A

   1. The WHNP/CNM looking after you

      a. Yes
      b. No

   2. The PMHNP for further management

      a. Yes

      b. No

Edinburgh Postnatal Depression Scale[1] (EPDS)

Name: Kendall Smith

Address: 382 Birch Streeet
Philadelphia, PA

Your Date of Birth: 32 years ago

Baby's Date of Birth: 3 weeeks ago

Phone: ____________________

As you are pregnant or have recently had a baby, we would like to know how you are feeling. Please check the answer that comes closest to how you have felt **IN THE PAST 7 DAYS**, not just how you feel today.

Here is an example, already completed.

I have felt happy:

☐ Yes, all the time
☒ Yes, most of the time
☐ No, not very often
☐ No, not at all

This would mean: "I have felt happy most of the time" during the past week. Please complete the other questions in the same way.

In the past 7 days:

1. I have been able to laugh and see the funny side of things
   - ☐ As much as I always could
   - ☐ Not quite so much now
   - ☒ Definitely not so much now
   - ☐ Not at all

2. I have looked forward with enjoyment to things
   - ☐ As much as I ever did
   - ☒ Rather less than I used to
   - ☐ Definitely less than I used to
   - ☐ Hardly at all

*3. I have blamed myself unnecessarily when things went wrong
   - ☐ Yes, most of the time
   - ☒ Yes, some of the time
   - ☐ Not very often
   - ☐ No, never

4. I have been anxious or worried for no good reason
   - ☐ No, not at all
   - ☐ Hardly ever
   - ☐ Yes, sometimes
   - ☒ Yes, very often

*5 I have felt scared or panicky for no very good reason
   - ☒ Yes, quite a lot
   - ☐ Yes, sometimes
   - ☐ No, not much
   - ☐ No, not at all

*6. Things have been getting on top of me
   - ☒ Yes, most of the time I haven't been able to cope at all
   - ☐ Yes, sometimes I haven't been coping as well as usual
   - ☐ No, most of the time I have coped quite well
   - ☐ No, I have been coping as well as ever

*7 I have been so unhappy that I have had difficulty sleeping
   - ☐ Yes, most of the time
   - ☒ Yes, sometimes
   - ☐ Not very often
   - ☐ No, not at all

*8 I have felt sad or miserable
   - ☒ Yes, most of the time
   - ☐ Yes, quite often
   - ☐ Not very often
   - ☐ No, not at all

*9 I have been so unhappy that I have been crying
   - ☐ Yes, most of the time
   - ☒ Yes, quite often
   - ☐ Only occasionally
   - ☐ No, never

*10 The thought of harming myself has occurred to me
   - ☐ Yes, quite often
   - ☐ Sometimes
   - ☒ Hardly ever
   - ☐ Never

Administered/Reviewed by Abigail Howe-Heyman, PhD, CNM, RN Date Today

[1]Source: Cox, J.L., Holden, J.M., and Sagovsky, R. 1987. Detection of postnatal depression: Development of the 10-item Edinburgh Postnatal Depression Scale. *British Journal of Psychiatry* 150:782-786 .

[2]Source: K. L. Wisner, B. L. Parry, C. M. Piontek, Postpartum Depression N Engl J Med vol. 347, No 3, July 18, 2002, 194-199

Users may reproduce the scale without further permission providing they respect copyright by quoting the names of the authors, the title and the source of the paper in all reproduced copies.

Edinburgh Postnatal Depression Scale[1] (EPDS)

Postpartum depression is the most common complication of childbearing.[2] The 10-question Edinburgh Postnatal Depression Scale (EPDS) is a valuable and efficient way of identifying patients at risk for "perinatal" depression. The EPDS is easy to administer and has proven to be an effective screening tool.

Mothers who score above 13 are likely to be suffering from a depressive illness of varying severity. The EPDS score should not override clinical judgment. A careful clinical assessment should be carried out to confirm the diagnosis. The scale indicates how the mother has felt ***during the previous week***. In doubtful cases it may be useful to repeat the tool after 2 weeks. The scale will not detect mothers with anxiety neuroses, phobias or personality disorders.

Women with postpartum depression need not feel alone. They may find useful information on the web sites of the National Women's Health Information Center <www.4women.gov> and from groups such as Postpartum Support International <www.chss.iup.edu/postpartum> and Depression after Delivery <www.depressionafterdelivery.com>.

SCORING

QUESTIONS 1, 2, & 4 (without an *)
Are scored 0, 1, 2 or 3 with top box scored as 0 and the bottom box scored as 3.

QUESTIONS 3, 5-10 (marked with an *)
Are reverse scored, with the top box scored as a 3 and the bottom box scored as 0.

Maximum score: 30
Possible Depression: 10 or greater
Always look at item 10 (suicidal thoughts)

Users may reproduce the scale without further permission, providing they respect copyright by quoting the names of the authors, the title, and the source of the paper in all reproduced copies.

Instructions for using the Edinburgh Postnatal Depression Scale:

1. The mother is asked to check the response that comes closest to how she has been feeling in the previous 7 days.

2. All the items must be completed.

3. Care should be taken to avoid the possibility of the mother discussing her answers with others. (Answers come from the mother or pregnant woman.)

4. The mother should complete the scale herself, unless she has limited English or has difficulty with reading.

[1]Source: Cox, J.L., Holden, J.M., and Sagovsky, R. 1987. Detection of postnatal depression: Development of the 10-item Edinburgh Postnatal Depression Scale. *British Journal of Psychiatry* 150:782-786.

[2]Source: K. L. Wisner, B. L. Parry, C. M. Piontek, Postpartum Depression N Engl J Med vol. 347, No 3, July 18, 2002, 194-199
